# Correction: The selective estrogen receptor downregulator GDC-0810 is efficacious in diverse models of ER+ breast cancer

**DOI:** 10.7554/eLife.44851

**Published:** 2019-01-07

**Authors:** James D Joseph, Beatrice Darimont, Wei Zhou, Alfonso Arrazate, Amy Young, Ellen Ingalla, Kimberly Walter, Robert A Blake, Jim Nonomiya, Zhengyu Guan, Lorna Kategaya, Steven P Govek, Andiliy G Lai, Mehmet Kahraman, Dan Brigham, John Sensintaffar, Nhin Lu, Gang Shao, Jing Qian, Kate Grillot, Michael Moon, Rene Prudente, Eric Bischoff, Kyoung-Jin Lee, Celine Bonnefous, Karensa L Douglas, Jackaline D Julien, Johnny Y Nagasawa, Anna Aparicio, Josh Kaufman, Benjamin Haley, Jennifer M Giltnane, Ingrid E Wertz, Mark R Lackner, Michelle A Nannini, Deepak Sampath, Luis Schwarz, Henry Charles Manning, Mohammed Noor Tantawy, Carlos L Arteaga, Richard A Heyman, Peter J Rix, Lori Friedman, Nicholas D Smith, Ciara Metcalfe, Jeffrey H Hager

Joseph JD, Darimont B, Zhou W, Arrazate A, Young A, Ingalla E, Walter K, Blake RA, Nonomiya J, Guan Z, Kategaya L, Govek SP, Lai AG, Kahraman M, Brigham D, Sensintaffar J, Lu N, Shao G, Qian J, Grillot K, Moon M, Prudente R, Bischoff E, Lee K-J, Bonnefous C, Douglas KL, Julien JD, Nagasawa JY, Aparicio A, Kaufman J, Haley B, Giltnane JM, Wertz IE, Lackner MR, Nannini MR, Sampath D, Schwarz L, Manning HC, Tantawy MN, Arteaga CL, Heyman RA, Rix PJ, Friedman L, Smith ND, Metcalfe C, Jeffrey HH. 2016. The selective estrogen receptor downregulator GDC-0810 is efficacious in diverse models of ER+ breast cancer. *eLife* **5**:e15828. doi: 10.7554/eLife.15828.

Published 13, July 2016

Due to an oversight at the time of the manuscripts publication a number of competing interests were omitted which we would like to declare:

Carlos L Arteaga (CLA) was a consultant for Genentech in 2015 and 2016. The consulting activities were not related to the manuscript. CLA is a member of the Komen Foundation’s Scientific Advisory Board.

Amy Young, Ellen Ingalla, Jim Nonomiya, Jennifer M Giltnane, Michelle A Nannini, and Deepak Sampath: Employed by Genentech and own shares.

The competing interest for Alfonso Arrazate was incorrectly listed in the original publication as ‘Shareholders of Seragon’ and has now been corrected to ‘Employed by Genentech and own shares.’

The article has been corrected accordingly.

