# Supplementary material for: The selective estrogen receptor downregulator GDC-0810 is efficacious in diverse models of ER+ breast cancer
Source: eLife. 2016 Jul 13;5:e15828. doi: 10.7554/eLife.15828 (PMC4961458; doi:10.7554/eLife.15828)
Supplement: Supplementary file 1. — (A) Radioligand binding assay (B) GDC-0810 nuclear hormone receptor reporter activity; agonist mode (C) GDC-0810 nuclear hormone receptor reporter activity; antagonist mode. [file elife-15828-supp1.docx]

**Supplementary File 1A: Radioligand Binding Assay**

| **Compound** | **GR** | | | | **AR** | | |
| --- | --- | --- | --- | --- | --- | --- | --- |
|  | **n** | **IC_50_ (nM)** | **Std Dev** | **RBA** | **n** | **IC_50_ (nM)** | **RBA** |
| Dexamethasone | 4 | 2.9 | 1.4 | 100.00 | ND | ND | ND |
| R1881 | ND | ND | ND | ND | 2 | 0.7 | 100 |
| GDC-0810 | 3 | 987 | 547 | 0.29 | 2 | > 4000 | < 0.02 |
| Fulvestrant | 3 | 817 | 318 | 0.35 | 2 | > 4000 | < 0.02 |
| 4-Hydroxytamoxifen | 3 | > 1800 | ND | < 0.158 | 2 | > 4000 | < 0.02 |

RBA = relative binding affinity

ND = not determined

**Supplementary File 1B: GDC-0810 NHR Transcriptional Reporter Activity: Agonist**

**Mode**

| **Treatment** | **GR** | | **MR** | | **PR-A** | | **PR-B** | |
| --- | --- | --- | --- | --- | --- | --- | --- | --- |
|  | **n** | **E_max_*** | **N** | **E_max_*** | **n** | **E_max_*** | **n** | **E_max_*** |
| RU-486 | 3 | 8.06 ±0.66 |  | ND | 4 | 13.29 ±10.23 | 3 | 3.25 ±1.17 |
| Spironolactone |  | ND | 3 | 6.24 ±0.37 |  | ND |  | ND |
| 4-OHT | 2 | 1.99 ±1.52 | 2 | 0.00 ±0.00 | 3 | 0.04 ±0.06 | 2 | 0.39 ±0.23 |
| GDC-0810 | 3 | 1.78 ±1.21 | 3 | 0.09 ±0.16 | 3 | 6.68 ±7.97 | 3 | 0.35 ±0.31 |
| Basal Activity | 3 | 1.71 ±1.28 | 3 | 1.40 ±0.82 | 4 | 18.01 ±8.58 | 3 | 0.44 ±0.26 |

*E_max_ is % of maximal dexamethasone- (for GR), aldosterone-stimulated (for MR), or R5020-stimulated (PR) activity.

ND = not determined.

**Supplementary File 1C: GDC-0810 NHR Transcriptional Reporter Activity: Antagonist Mode**

| **Treatment** | **GR** | | | **MR** | | | **PR-A** | | | **PR-B** | | | |
| --- | --- | --- | --- | --- | --- | --- | --- | --- | --- | --- | --- | --- | --- |
|  | **n** | **E_max_*** | **IC_50_**  **(μM)** | **n** | **E_max_*** | **IC_50_**  **(μM)** | **n** | **E_max_*** | **IC_50_**  **(μM)** | **n** | **E_max_*** | **IC_50_**  **(μM)** |  |
| RU-486 | 3 | 5.84  ±2.78 | 0.006  ±0.004 |  | ND | ND | 4 | 18.30  ±5.52 | 0.0004  ±0.0002 | 3 | 3.19  ±0.88 | 0.002  ±0.003 |  |
| Spironolactone |  | ND | ND | 3 | 7.27  ±1.17 | 0.0011 ±0.010 |  | ND | ND |  | ND | ND |  |
| 4-OHT | 2 | 76.43  ±8.61 | 1.47  ±1.18 | 2 | 0.00 | 6.42 ±0.75 | 3 | 6.80  ±11.78 | 12.0  ±4.5 | 2 | 0.00 | 7.16  ±2.15 |  |
| GDC-0810 | 3 | 48.28  ±43.42 | 20.7  ±28.7 | 3 | 0.00 | 9.62 ±6.64 | 4 | 18.44  ±14.49 | 4.2±4.1 | 3 | 0.00 | 3.75  ±1.21 |  |
| Basal Activity | 3 | 2.09  ±1.01 | NA | 3 | 1.48  ±0.81 | NA | 4 | 18.10  ±8.69 | NA | 3 | 1.14 ±1.05 | NA |  |

E_max_ is % of maximal dexamethasone (10 nM, GR), aldosterone (1 nM, MR) and R5020 (1 nM, PR)-stimulated activity. With the exception of the vehicle, assays were run in the presence of the appropriate agonist (10 nM dexamethasone, GR; 1 nM aldosterone, MR; 1 nM R5020, PR).

NA = not applicable; ND = not determined
