# Supplementary material for: The selective estrogen receptor downregulator GDC-0810 is efficacious in diverse models of ER+ breast cancer
Source: eLife. 2016 Jul 13;5:e15828. doi: 10.7554/eLife.15828 (PMC4961458; doi:10.7554/eLife.15828)
Supplement: Supplementary file 2. — (A) GDC-0810 mouse pharmacokinetics (B) Fulvestrant plasma concentrations. [file elife-15828-supp2.docx]

**Supplementary File 2A: GDC-0810 Mouse Pharmacokinetics**

| **Compound** | **Dose (mg/kg/day)** | **C_last_ (µg/mL)** | **T_1/2_ (hr)** | **AUC_0-t_ (µg·hr/mL)** | **C_max_  (µg/mL)** | **T_max_ (hr)** |
| --- | --- | --- | --- | --- | --- | --- |
| GDC-0810 | 10 | 0.007 | 8.0 | 5.5 | 1.637 | 1.0 |
|  | 30 | 0.013 | 4.0 | 21.5 | 5.333 | 1.0 |
|  | 100 | 0.216 | 3.0 | 94.1 | 9.313 | 8.0 |

| **Compound** | **CL**  **(mL/min/kg)** | **V_ss_ (L/kg)** | **t_1/2_  (hr)** | **C_max_ (Oral) (µg/mL)** | **AUC_0-inf_ (Oral) (µg·hr/mL)** | **Oral**  **%F** |
| --- | --- | --- | --- | --- | --- | --- |
| GDC-0810 | 11 | 1.2 | 2.6 | 4.4 | 8.8 | 61 |

**Supplementary File 2B: Fulvestrant Plasma Concentrations**

| **Compound** | **Regimen** | | **Day** | **Time (hr)** | **Plasma Conc (µg/mL)** | |
| --- | --- | --- | --- | --- | --- | --- |
|  | **Frequency** | **Dose (mg/kg)** |  |  | **Mean** | **SD** |
| Fulvestrant | 3x/wk | 200 | 57 | 0 | 0.665 | 0.236 |
| Fulvestrant | 3x/wk | 200 | 28 | 0 | 0.731 | 0.7128 |
| Fulvestrant | Days 1, 3 & 8 Days 15 & 22 | 50 25 | 21 | 0 | 0.015 | 0.009 |
