# Supplementary material for: The selective estrogen receptor downregulator GDC-0810 is efficacious in diverse models of ER+ breast cancer
Source: eLife. 2016 Jul 13;5:e15828. doi: 10.7554/eLife.15828 (PMC4961458; doi:10.7554/eLife.15828)
Supplement: Supplementary file 3. — (A) Transcriptional Real-time PCR Oligonucleotide Sequence (B) ER-ChIP Real-time PCR Oligonucleotide Sequence. [file elife-15828-supp3.docx]

**Supplementary File 3A. Transcriptional Real-time PCR Oligonucleotide Sequence**

| **Gene** | **Forward Primer Sequence** | **Reverse Primer Sequence** |
| --- | --- | --- |
| **RAB37** | TTCATGAGTATGCCCAGAGG | GATCACTCTTTCGCTGCTCA |
| **GPR56** | GCTGTGCATAGGACTCCAGA | CCAGGTTGGTGATGTAGCTG |
| **DDR1** | AAGATCTTACGGCCAGATGC | TGAGCCTCGACATGATCTTC |
| **INHBB** | GAGAAGAGGGTGGACCTCAA | GCTGTCACACTGCACGTCTA |
| **AGR2** | GGTGACCAACTCATCTGGACTC | TGACTGTGTGGGCACTCATCCA |
| **SNX24** | CTTGCCCTCTCTACCAAAGG | CTGCAGGCAAGACATATGGA |
| **STC1** | GGTGCAGGAAGAGTGCTACA | AGGCTTCGGACAAGTCTGTT |
| **SLC9A3R1** | GGACAGGGAAACTGACGAGT | TTCACGACTGTTCTCCTTCTG |
| **KRT15** | GACATAAAGACACGGCTGGA | CTGAAGAGGCTTCCCTGATG |
| **SLC2A1** | TCATCGTGGCTGAACTCTTC | ACCACACAGTTGCTCCACAT |
| **KCNK6** | CTCTCTTCTTCGCCAGCAC | TCAGTCAGTGGCGTTGTGTA |
| **LRP2** | TGTTTCAATGCTGTCTGCAA | GGACACATCCACATCCACAT |
| **ERBB2** | GGAAGTACACGATGCGGAGACT | ACCTTCCTCAGCTCCGTCTCTT |
| **RFTN1** | ACATGGCTTGACAGATGGAG | GGCACGTAGTCTGTCTGCAC |
| **MAOB** | CTTGCAGAAGAGTGGGACAA | TCACAAAGAGAGTGGCAAGC |
| **RASGRP** | GGAGGCTAACAAGGACTTGGTAC | GGTGGCTTTGAAGGTGTTAGTGG |
| **MKI67** | CCTGCTACTCCAAAGAAGCC | TGGTACAAGGAGAGTTTGCG |
| **HSPB8** | TCAAGCCAGAGGAGTTGATG | GCCACCTTCTTGCTGTTTCT |
| **RHOBTB** | GGAATTGGCTCAGTTTCACA | CCTTACGGAACTTGGAGCAT |
| **MUC1** | CCTACCATCCTATGAGCGAGTAC | GCTGGGTTTGTGTAAGAGAGGC |
| **PDZK1** | CCTTTCTCAAGGAATGAGTTGTG | CCGCCTGTAAGACAAATGATAAC |
| **BLNK** | CCCCTACCCAGCTTTTCATCT | TCACAGGCTCCAGCATACCA |
| **WISP2** | CTGTATCGGGAAGGGGAGAC | GGGAAGAGACAAGGCCAGAA |
| **PGR** | GTCAGTGGGCAGATGCTGTA | AGCCCTTCCAAAGGAATTGT |
| **NELL** | TTGAGTCCTGGATAGACGGCTG | TGCCATCCACATACGCAAGAGC |
| **ABCC5** | GGCTGTATTACGGAAAGAGGCAC | TCTTCTGTGAACCACTGGTTTCC |
| **pS2/TFF1** | TTGTGGTTTTCCTGGTGTCA | CCGAGCTCTGGGACTAATCA |
| **GREB1** | GGCAGGACCAGCTTCTGA | CTGTTCCCACCACCTTGG |
| **MYBL1** | CGTGGAGGCAAACGCTGTGTTA | GGTGGATTTGATAGGAGAAGCAG |

**Supplementary File 3B. ER-ChIP Real-time PCR Oligonucleotide Sequence**

| **Gene** | **Forward Primer Sequence** | **Reverse Primer Sequence** |
| --- | --- | --- |
| **GREB ENHANCER 3** | GAAGGGCAGAGCTGATAACG | GACCCAGTTGCCACACTTTT |
| **GREB ENHANCER 5** | GCCCAGGAGACAGGTTGTAA | TATGACTCTTGGCCCTGTCC |
| **PGR ENHANCER 1** | GCCTGACCTGTTGCTTCAAT | GCAGGACGACTTCTCAGACC |
| **TFF1 PROMOTER** | GGCCATCTCTCACTATGAATCACTTCTGC | GGCAGGCTCTGTTTGCTTAAAGAGCG |
| **WISP2 ENHANCER** | GCTTGACCCCATCATCTAGCGGTGC | GGTGTGACCCAGAGCAAAACTTCCC |
| **MYC ENHANCER** | ACTCTGCACTGCCAGACAAA | TGGAAACCACATTTTGGTCA |
| **ESR1 ENHANCER 3** | GAAACAGCCCCAAATCTCAA | TTGTAGCCAGCAAGCAAATG |
| **GATA3 ENHANCER 3** | ATTCTTGTGCATGGTGCTGA | TTACAGGGCAAAAAGGTTGG |
| **CTSD ENHANCER 1** | GCCACAGGCAGCTTTAGTTC | CATTCACAGCCTCCACCTTT |
| **CTSD ENHANCER 2** | GCTAACATGTTGCCTGCTCA | TTCATATCTACCGCCCAAGG |
